# Diagnostic value of neutrophil gelatinase-associated lipocalin (NGAL) as an early biomarker for detection of renal failure in hypertensives: a case–control study in a regional hospital in Ghana

**DOI:** 10.1186/s12882-023-03120-6

**Published:** 2023-04-26

**Authors:** Mark Danquah, William K. B. A. Owiredu, B. A. Eghan Jnr, Dorcas Serwaa, Enoch Odame Anto, Maxwell Owusu Peprah, Christian Obirikorang, Linda A. Fondjo

**Affiliations:** 1 Department of Medical Laboratory Technology, Faculty of Applied Science and Technology, Sunyani Technical University, Sunyani, Ghana; 2 Department of Pharmaceutical Sciences, Faculty of Applied Science and Technology, Sunyani Technical University, Sunyani, Ghana; 3 Department of Molecular Medicine, School of Medicine and Dentistry, Kwame Nkrumah University of Science and Technology, Kumasi, Ghana; 4 Department of Molecular Medicine, School of Medicine and Dentistry, Kwame Nkrumah University of Science and Technology, Ghana/Komfo Anokye Teaching Hospital, Kumasi, Ghana; 5 Department of Obstetrics and Gynecology, University of Melbourne, Melbourne, Australia; 6 Department of Medical Diagnostics, Faculty of Allied Health Sciences, Kwame Nkrumah University of Science and Technology, Kumasi, Ghana; 7 Department of Nursing and Midwifery, Presbyterian Nursing and Midwifery, Dormaa Ahenkro, Ghana

**Keywords:** Hypertension, Renal failure, Creatinine, Neutrophil gelatinase associated lipocalin

## Abstract

**Background:**

Renal failure is one of the most serious vascular effects of hypertension. For better therapy and prevention of complications, early kidney disease identification in these patients is absolutely essential. However, current studies have proposed plasma Neutrophil Gelatinase Associated Lipocalin (pNGAL) to be a better biomarker comparative to serum creatinine (SCr). This study assessed the diagnostic utility of plasma neutrophil gelatinase-associated lipocalin (pNGAL) as a biomarker for early nephropathy diagnosis in hypertensive individuals.

**Methods:**

This hospital-based case–control study comprised 140 hypertensives and 70 healthy participants. A well-structured questionnaire and patient case notes were used to document relevant demographic and clinical information. 5 ml of venous blood sample was taken to measure fasting blood sugar levels, creatinine, and plasma NGAL levels. All data were analyzed using the Statistical Package for Social Sciences (SPSS release 20.0, copyrite©SPSS Inc.) and a *p*-value < 0.05 was considered statistically significant.

**Results:**

In this study the plasma neutrophil gelatinase-associated lipocalin (NGAL) levels were significantly higher in cases compared to controls. Hypertensive cases also had significantly higher waist-circumference compared to the control group. The median fasting blood sugar level was significantly higher in cases compared to controls. This study established the use of Modification of Diet in Renal Disease (MDRD), Chronic Kidney Disease Epidemiology Collaboration (CKD-EPI), and Cockcroft and Gault formula (CG) as the most accurate predictive equations for assessing renal dysfunction. The threshold for NGAL above which renal impairment can be assessed was found to be 109.4 ng/ml (sen-91%, spec. – 68%), 120 ng/ml (sen- 100%, spec- 72%) and 118.6 ng/ml (sen- 83%, spec- 72%) for MDRD, CKD-EPI and CG equations respectively. The prevalence of CKD was 16.4%, 13.6% and 20.7% respectively using the MDRD, CKD-EPI and CG.

**Conclusion:**

From this study, pNGAL is a better indicator of kidney impairment in the early stages of CKD as compared with sCr in general hypertensive population.

## Introduction

Kidney disease is a diverse range of illnesses that affects kidney structure and function and could lead to renal failure [1]. Acute Kidney Injury (AKI) characterizes kidney dysfunction with glomerular filtration rate of < 60 ml/min/1.73m^2^ for less than three months or an increase in serum creatinine by > 50% for < 3 months whilst Chronic Kidney Disease (CKD) defines kidney dysfunction with decreased estimated glomerular filtration rate (eGFR) and albuminuria for at least three months [2]. So, the overall renal function (glomerular filtration rate, GFR) and the existence of kidney damage determined by kidney biopsy or other markers of kidney damage are used to assess kidney disease [3].

According to reports by Abd EIHafeez et al. [4], the prevalence of chronic kidney disease among the African population ranges from 2 to 41%, with a combined estimate of 16.5% in West/Central-West Africa. By 2030, it is predicted that sub-Saharan African region would have a significantly high incidence of kidney disease due to the wide range of potential causes of the condition [5]. Over the past twenty years it has been established that early detection and treatment of kidney disease can prevent kidney disease progression [6].

CKD is known to be linked to aging, diabetes, hypertension, obesity, and other cardiovascular diseases; the presumptive pathological findings are diabetic glomerulosclerosis and hypertensive nephrosclerosis [7]. After diabetes mellitus, hypertensive kidney disease is the second most common cause of end-stage renal disease (ESRD) characterized by reduced renal function and/or albuminuria [8]. In Africa, hypertension and its complications including renal dysfunction have been reported and constitute a major factor in the high morbidity and mortality among adults in the sub-Saharan Africa [9]. Studies have also proposed that higher blood pressure (systolic blood pressure ≥ 140 mmHg; diastolic blood pressure ≥ 90 mmHg) predicts more liberal development of nephropathy, and proteinuria is a well-recognized major risk factor for the development of nephropathy in people with essential hypertension [10]. It is unknown how prevalent kidney disease is in the Ghanaian population but Osafo et al. [11] posited that the prevalence of chronic kidney disease (CKD) was 46.9% among hypertensive individuals in Ghana.

Although blood creatinine is commonly used as an index of renal function, higher creatininemia is primarily a marker of glomerular filtration [12] and cannot be considered an ideal biomarker for the estimation of kidney injury, because it is insensitive and is influenced by muscle mass, gender, race, and medications and is unreliable for the diagnosis of renal tubular injury in the absence of significant reduction in the glomerular filtration rate (GFR) [13]. Moreover, since serum creatinine (SCr) is produced in the liver and is immediately impacted by hepatic parenchymal dysfunction, it cannot accurately reflect GFR in a number of disorders [14]. Therefore, a biomarker of kidney damage that is able to indicate the presence of both early damage and identify patients at an increased risk of progressive disease would impact kidney disease diagnosis and treatment. Neutrophil Gelatinase-Associated Lipocalin (NGAL) has been proposed to be useful for the diagnosis of CKD [15] and it has the potential to be an ideal biomarker in the early detection of CKD. This tiny secreted glycoprotein of 25 kDa also known as lipocalin-2, 24p3, siderocalin, or uterocalin is discovered in mature neutrophil granules and expressed in a wide variety of cells both in cardiomyocytes and renal endothelial cells [16]. Furthermore, plasma NGAL (pNGAL) assessment for the early detection of renal failure in hypertensive adult population in Ghana has not been explored. Therefore, this study examined the predictive accuracy of pNGAL measurement for detecting CKD in comparison with the conservative biomarker creatinine, evaluated the specificity and sensitivity of NGAL in the prediction of chronic kidney diseases and also evaluated the diagnostic efficiency of NGAL in comparison with validated creatinine base renal equations.

## Materials and methods

### Study design and study setting

This hospital-based case–control study was conducted at the Bono regional hospital in Sunyani Municipal. The Bono region can be found in the middle belt of Ghana which is bordered to the north by the Black Volta River and to the east by the Lake Volta, and to the south by the Ashanti, Eastern and Western regions. Its capital city is Sunyani and its Municipal Assembly covers a total land area of 506.7 km2. It lies between Latitudes 70 20’N and 70 05’N and Longitudes 20 30’W and 20 10’W. The hospital is a 300-bed capacity with a special hypertensive clinic. The Bono regional hospital is the main referral hospital in the municipality which serves all the nearby districts and villages.

#### Sample size determination

The necessary sample was obtained using the Kelsey’s formula:

$$N_{cases-Kelsey}=\left[\frac{r+1}r\right]\frac{P(1-P)\left(\frac{Z_\alpha+Z_\beta}2\right)^2}{\left(\mathrm p1-\mathrm p2\right)^2},\;\mathrm{and}\;\mathrm P=\left[\frac{p1+(rXp2)}{r+1}\right]$$

Where r is the ratio of Hypertensives to healthy controls, which is 2:1 in this study, ${Z}_{\frac{\alpha }{2}}$ represents the critical value of the normal dispersion at α/2 (For this study at a confidence interval of 95%, α is 0.05and the critical value is 1.96), Zβ represents the critical value of the normal distribution at β (this study used a power of 80%, β is 0.2 and the critical value is 0.84). p1 represents the percentage of hypertensives with CKD in Ghana, which is 46.9%, p2 is the percentage of CKD in the control group, which is 13.3% according to Asare-Anane et al., 2013 [17]. And p1-p2 is the smallest difference in proportions that is clinically important.

From the formula above, the minimum number of hypertensives required for this study was 40 with 20 healthy controls. However this study employed 210 participants. 140 hypertensive patients and 70 health controls.

#### Study participants

This study enrolled 210 participants consisting of one hundred and forty (70 men and 70 women) hypertensives under management at the clinic, mean age of 59.23 ± 5.8 and seventy (38 men and 32 women) study participants without hypertension condition, with mean age of 56.49 ± 7.83 as controls. This study excluded participants with chronic diseases such as liver disease, diabetes, elevated serum creatinine and urea, HIV, TB and hepatitis B and C to help minimize potential confounding factors. All participants were enrolled regardless of their educational or marital status and patients’ history was carefully recorded by interview and confirmed from their folders as well.

#### Data collection

A well-structured questionnaire and patients’ medical records were used to document relevant demographic and clinical history of the participants. Hypertensive patients were diagnosed according to the WHO criteria of systolic blood pressure ≥ 140 mmHg; diastolic blood pressure ≥ 90 mmHg and confirmed on two occasions by a physician. Height to the nearest centimeter without shoes and weight to the nearest 0.1 kg in light clothing was estimated. Weight measurement was done using a bathroom scale (Zhongshan Camry Electronic Co. Ltd, Guangdong, and China) and height was measured with a wall-mounted ruler. Waist circumference measurements were taken using Constant tension tape such as, Figure Finder Tape Measure and was measured in the nearest centimeter. The blood pressure of the study participants was measured by qualified health personnel using a mercury sphygmomanometer and a stethoscope in accordance with the recommendations of the American Heart Association.

#### Blood sampling and analysis

A fingertip capillary whole blood sample was collected from each subject after overnight fasting for determination of fasting blood sugar levels using Accu-Chek Advantage Blood Glucose Monitoring System. (AC; 3 Roche Diagnostics, Indianapolis, IN). Calibration of the instrument was performed at 7:00 am using the test kit glucose control solution. Vacutainer needle was used to draw all the two set of blood samples, with 3mls in the vacutainer plain tube and 2mls in a chilled vacutainer tube containing potassium ethylenediamine tetracetate (EDTA). The plain tube sample after clotting was centrifuged and the serum was stored at—80 °C until assayed and the chilled EDTA sample was promptly separated by a refrigerated centrifuge (at 4c, 5 min, 5000 rpm) and the plasma was stored at—80 °C until further analysis according to standard operating procedures.

#### Laboratory assay (Creatinine, NGAL)

Creatinine levels were measured on serum samples using a BT 3000 fully automated chemistry analyzer (BT-3000) following the standard operating procedures for estimating quantitative analyte. The accepted reference range for creatinine measurement was 0.74 to 1.35 mg/dL or (65.4 to 119.3 micromoles/L) for adult men, and 0.59 to 1.04 mg/dL or (52.2 to 91.9 micromoles/L) for adult women. NGAL measurement was done using commercially available ELISA kit (Bioverder, Norway). According to the manufacturer's instructions, reagent was used to measure samples from both the subjects and the controls using the solid phase ELISA method. The accepted reference range in the laboratory was 0.625 ng/ml – 20 ng/ml.

#### Statistical analysis

The data obtained from the study was entered into Microsoft excel software and analyzed using the statistical Package for Social Sciences (SPSS release 20.0, copyrite©SPSS Inc.) Continuous variables with normal distribution were expressed as mean ± standard deviation (SD) and the median and inter quartile range (IQR) used for variables that were not normally distributed. Categorical variables were expressed in proportions and compared with chi-square test. Receiver Operating Characteristics Curve was used to evaluate an area under the curve of NGAL in the assessment of renal impairment. Bland and Altman plots were used to assess bias among the various creatinine equations (MDRD, CKD-EPI AND CG). Confidence interval of 95% was used and a *P*-value of < 0.05 was considered to be statistically significant.

## Results

From Table 1 below, the median systolic blood pressure, diastolic blood pressure and mean arterial pressure were significantly higher in the cases than the controls (*p* < 0.0001). The median weight and waist circumference were significantly higher in cases than the controls. Similarly, the biochemical parameters including the median fasting blood sugar and plasma neutrophil gelatinase-associated lipocalin (NGAL) were significantly higher in cases compared to controls (*p* < 0.05). However, there was no significant difference of median creatinine levels between cases and control (*p* > 0.05). For the chronic kidney diseases equation, no significant differences were observed between median MDRD equation and CKD-EPI equation values of cases and controls (*p* > 0.05).

**Table 1:** Comparison of age, hemodynamic indices, anthropometric indices biochemical parameters and chronic kidney disease equations between cases and control

**Variables**	**Cases (*n* =)** Median, IQR	**Control *n* ( =)** Median, IQR	***P*-value**
Age (years)	59.0(55.0–62.0)	58.0(55.0–60.0)	0.051
SDP(mmHg)	150.0(145.0–160)	119.0(111.0–120.0)	** < 0.0001**
DBP(mmHg)	99.0 (94.0–101.0)	70.0(65.0–80.0)	** < 0.0001**
MAP (mmHg)	115.3(111.0–160.0)	86.0(82.0–89.6)	** < 0.0001**
Weight (kg)	67.5(63.0–72.75)	63.0(58.0–69.0)	**0.0021**
Waist Circumference (cm)	80.5(65.0–72.7)	65.0(55.0–77.0)	** < 0.0001**
FBG (mmol/l)	4.5(4.0–5.1)	3.8(3.2.4.4)	** < 0.0001**
Creatinine (µmol/l))	0.90(0.77–1.16)	0.88(0.77–0.97)	0.192
CKD-EPI equation	85.5(71.0–111.8)	98.0(79.0–110.0)	0.246
MDRD	81.5(69.0–111.0)	97.0(78.0–109.0)	0.180
NGAL (ng/ml)	23.5(9.7–160.4)	12.8(10.5–16.0)	**0.003**
Cockroft-Gault	72.6(61.9–87.6)	75.4(64.8–88.4)	0.882

*IQR* inter quartile range, *SBP* systolic blood pressure, *DBP* diastolic blood pressure, *FBG* Fasting Blood Glucose, *NGAL* neutrophil- gelatinase-associated lipocalin, *CKD* chronic kidney disease, *MDRD* Modification of Diet in Renal Disease

The diagnostic performance of NGAL in the assessment of renal impairment among hypertensive patients is shown in Table 2 below. The cut-off value for NGAL above which renal impairment would be diagnosed was 109.4ngml based on MDRD chronic kidney disease equation. On the basis of this threshold, the sensitivity, and specificity, positive predictive value and negative predictive value were 0.91, and 0.68, respectively. The cut-off value for NGAL above which renal impairment would be diagnosed was 120.0ngml based CKD-EPI creatinine-based equation. The sensitivity and specificity of NGAL for renal impairment diagnosis were 1.00 and 0.72respectively based on this cut-off values. The threshold for NGAL above which renal impairment would be diagnosed was 118.6ngml. On the basis of this threshold; the sensitivity and specificity of ultrasound for renal impairment diagnosis were 0.83 and 0.72 respectively.

**Table 2:** Diagnostic performances of NGAL in the assessment of renal impairment using MDRD, CKD-EPI creatinine and Cockroft-Gault eGFR equations

Variables	Sensitivity (95%CI)	Specificity (95%CI)	PPV	NPV	TP	TN	FP	FN	Youden Index	Associated Criterion
		Based on MDRD Equation								
NGAL	0.91(0.72–0.98)	0.68(0.59–0.76)	0.36	0.97	21	80	37	2	0.60	109.4
		Based on CKD-EPI Equation								
NGAL	1.00(0.80–1.00)	0.72(0.63–0.79)	0.36	1.00	19	87	34	0	0.72	120.2
		Based on Cockroft-Gault Equation								
NGAL	0.83(0.65–0.93)	0.72(0.63–0.79)	0.43	0.94	24	80	31	5	0.55	118.6

*PPV* positive predictive value, *NPV* negative predictive value, *TP* True Positive, *TN True* Negative, *FP* False Positive, *FN-False* Negative

Table 3 shows the partial correlation coefficients of hemodynamic indices, anthropometric indices, and biochemical parameters adjusted for age and gender. Among the participants with hypertension, increasing creatinine significantly associated increasing levels of neutrophil gelatinized-associated lipocalin (NGAL) after adjusting for age and gender. Waist Circumference was also found to be associated with increased levels of creatinine, and NGAL. In general, positive additive change in the renal impairment indices (NGAL, Creatinine) were significantly associated with corresponding increases in systolic blood pressure, diastolic blood pressure, mean arterial pressure and fasting blood sugar levels, among the hypertension subpopulation after adjusting for age and gender.

**Table 3:** Partial correlation coefficients hemodynamic indices, anthropometric indices, biochemical parameters adjusted for age and gender

Variables		Diastolic	MAP	Weight	WC	Height	FBS/RBS	Creatinine	NGAL
Systolic	r	0.49	0.85	0.26	0.45	-0.014	-0.03	0.48	0.57
	p	** < 0.0001**	< **0.0001**	**0.002**	** < 0.0001**	0.87	0.710	** < 0.0001**	** < 0.0001**
Diastolic	r		0.879	0.16	0.20	-0.096	-0.06	0.31	0.29
	p		** < 0.0001**	0.06	**0.017**	0.270	**0.470**	** < 0.0001**	**0.001**
Mean Arterial Pleasure	r			0.24	0.37	-0.066	-0.06	0.453	0.49
	p			0.004	< 0.0001	0.450	0.520	** < 0.0001**	** < 0.0001**
Weight	r				0.40	-0.134	0.14	0.34	0.333
	p				** < 0.0001**	**0.120**	0.097	** < 0.0001**	** < 0.0001**
Waist Circumference	r					0.041	0.086	0.69	0.76
	p					0.637	0.317	** < 0.0001**	** < 0.0001**
Height	r						0.065	-0.09	-0.105
	p						0.456	0.300	0.220
FBS/RBS	r							-0.017	0.099
	p							0.840	0.250
Creatinine	r								0.84
	p								** < 0.0001**
NGAL	r								
	p								

**Fig. 1:**
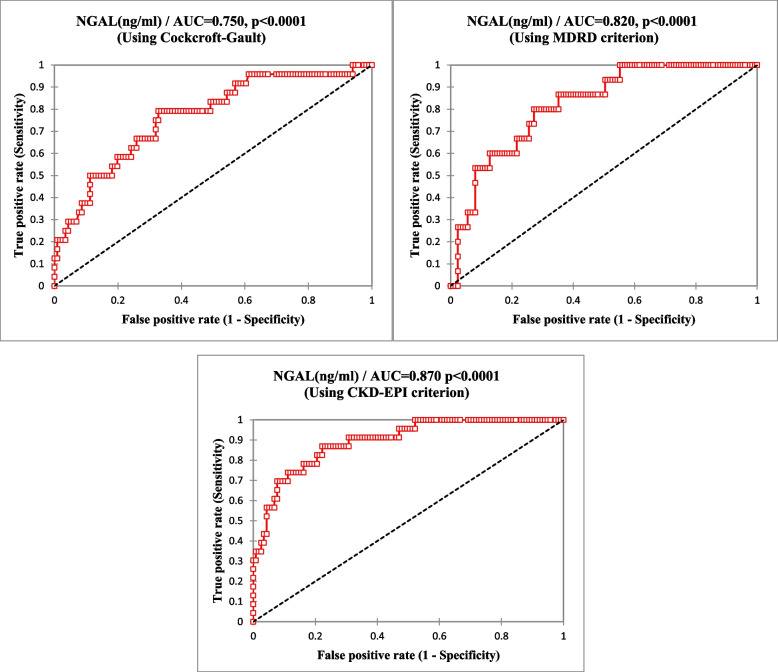
Receiver Operating Characteristic (ROC) of NGAL in the assessment of renal impairment among hypertensive

Figure 1 Shows the receiver operating characteristic curve depicting the area under curve of NGAL using the eGFR as the gold standard method (cut-off value of < 60mlmin). ROC analysis showed that NGAL had an AUC of 0.75(0.65–0.85) using Cockroft-Gault equation as gold standard method, 0.87(0.81–0.93) using CKD-EPI equation as gold standard method, and 0.82(0.73–0.90) using MDRD equation as gold standard method. Significant asymptomatic *p*-values were observed in all ROC analysis of NGAL for the different gold standard methods (*p* < 0.0001)

**Fig. 2:**
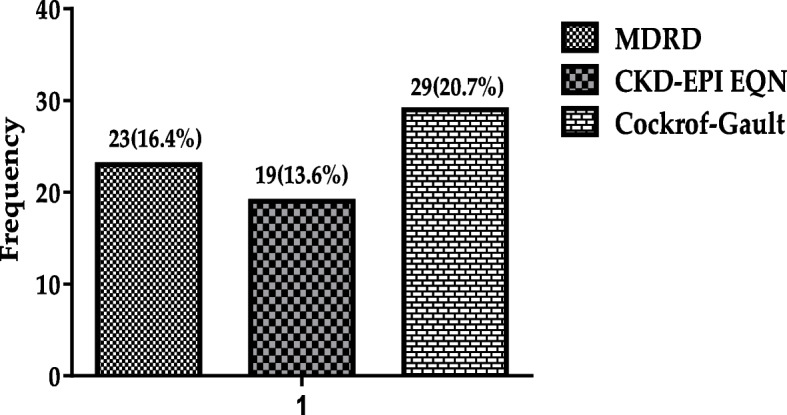
Prevalence of chronic kidney disease (CKD) based on MDRD equation, CKD-EPI creatinine, and Cockroft-Gault equation

As shown in Fig. 2, the prevalence of chronic kidney disease (CKD) based on MDRD equation was 16.4%, CKD-EPI creatinine equation was 13.6%, and Cockroft-Gault equation was 20.7% respectively.

**Fig. 3:**
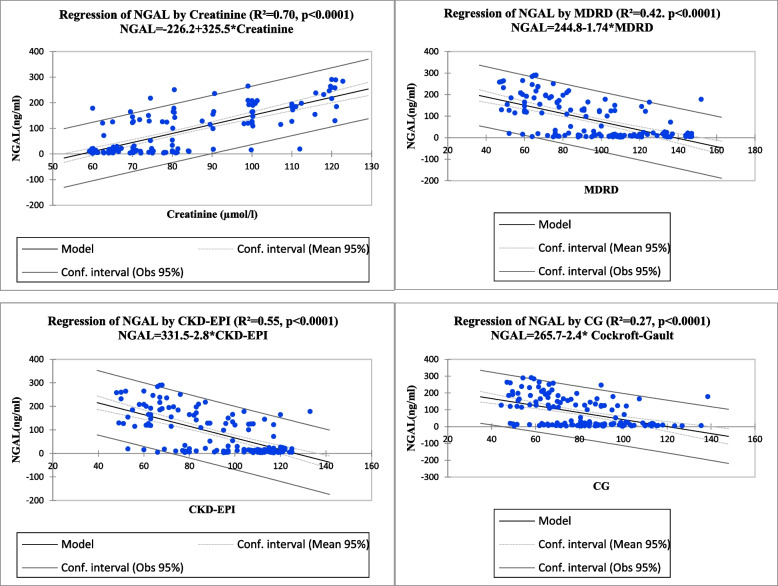
Scatterplot with linear regression analysis showing the relationship of Creatinine, MDRD equation, Cockroft-Gault equation and CKD-EPI creatinine equation with neutrophil gelatinized-associated lipocalin (NGAL) among hypertensive

Figure 3 shows, the regression plot of the individual patient data points for NGAL in relation to the creatinine showed a strong statistically significant positive relationship (*p* < 0.0001). The coefficient of determination (R^2^ value) was 0.70. This means that, each unit increase of NGAL will influence 70.0% of the increase change in the creatinine levels. NGAL was significant negatively related with MDRD equation (*p* < 0.0001), Cockroft-Gault equation (*p* < 0.0001) and CKD-EPI creatinine equation (*p* < 0.0001). The coefficients of determination (R^2^ = value) were 0.42, 0.55 and 0.27which means that, each unit decrease of NGAL influences 42.0%, 55.0% and 27.0% increase change in values of MDRD equation, Cockroft-Gault equation and CKD-EPI creatinine equations.

## Discussion

The search for more prognostically reliable, sensitive, and specific biomarkers to help manage patients with or at risk for renal disease has increased in recent years. The complications associated with chronic kidney disease and renal failure can be avoided or reduced with early detection and treatment [18]. Hypertension is known to be one of the risk factors for developing kidney disease [19]. Neutrophil gelatinase-associated lipocalin (NGAL) has recently received attention as a promising biomarker for the early diagnosis of renal failure. This hospital-based case control study examined the diagnostic value of plasma NGAL and compared it with serum creatinine in hypertensive patients.

Findings of this current study revealed higher NGAL figures in cases than the controls and there was no significant difference of median creatinine levels of cases compared to controls. Similar to this current study, Gharishvandi et al. [20] & Bolignano et al. [21] who assessed the diagnostic accuracy of NGAL and compared to creatinine in hypertensive patients had higher NGAL values among the cases compared to the controls. Malyszko et al. [22] also reported substantially lower NGAL in normotensives than in hypertensives with eGFR (MDRD and Cockcroft-Gault formulae) considerably greater in normotensives than in hypertensives despite comparable serum creatinine levels. Buonafine et al. [16] explained that, NGAL is quickly released in response to tubular injury which has led to its description as an acute renal lesion biomarker. They suggested that, urine NGAL concentrations can also be assessed and this makes it a preferred biomarker for clinical usage, along with its high stability and resistance to proteases. Plasma levels of creatinine are frequently used to assess renal function but an increasing number of studies report that NGAL is a more accurate indicator of acute renal injury (AKI).

The results of this current study also confirm the assertion by the National Kidney Foundation that high blood pressure (BP) is a common cause of CKD and hence patients with high BP are at an increased risk of loss of kidney function and development of CKD. A research on adult patients revealed that those who later developed clinically significant Acute Kidney Injury had significantly greater urine NGAL levels at 1, 3, and 18 h following heart surgery [23]. Also, song et al. [24] concluded that, higher levels of circulating NGAL correlates with high blood pressure and insulin resistance based on the role of NGAL-in oxidative stress, endothelial dysfunction, inflammation, and hypertension. In monitoring hypertensive patients, there is need for assessment of the presence of kidney disease especially those with decreased GFR as recommended by National Kidney Foundation [2].

The results of this current study indicated higher waist circumference in the cases compared to the controls. The increased waist circumference (WC) observed when the CKD subjects were compared to the controls is similar to the work of Levey et al. [2]. Indeed, central obesity, as evaluated by the waist circumference, has been indicated as an independent risk factor for renal dysfunction [25–28].

In this current study, the best cut-off values to predict renal impairment in hypertensive patients was assessed using all the three validated creatinine based equations and the best threshold for NGAL above which renal impairment would be diagnosed was 120.0ngml based on the CKD-EPI creatinine based equation which also had sensitivity and specificity of 100% and 72%.

The findings of this study are in conformity with the work of Levey et al., (2009) and also David Bolignano et al. with cut-off level for sNGAL of 435 ng/ml (sensitivity 83%, specificity 53.8%) [21, 29]. Again our cut-off values were slightly higher the study by Gharishvandi et al., 2014 pNGAL value of 32.2 ng/ml (sensitivity of 96% and specificity of 100%).

Our cut-off values were slightly lower than the findings of David Bolignano et al. because most of our patients were probably at the early stages of CKD [21].

The practical application of GFR predictive formula is to diagnose and stratify chronic kidney disease in patients with kidney disease. According to the study of Levey et al., [2] the CKD-EPI and the MDRD equations show better diagnostic accuracy than the CG formula as they do not require body weight. This, however, appears not to be the case in this current study in which all the three equations had good specificity values to detect GFR values less than 60 ml/min/1.73m^2^., although CKD-EPI was better compared to the other two, which is in direct conformity with the previous study.

The ROC curve showing the area under curve of NGAL in the assessment of renal impairment among hypertensive were assessed using GFR as the Gold standard. Significant asymptomatic p-value was obtained in all ROC analysis of NGAL for the different gold standard methods, but the best AUC was 0.87 (0.81–0.93) using CKD-EPI. The findings of this current study was consistent with the work of [20] who had AUC of _p_NGAL of 0.92 (0.87–0.98).

In this current study scatterplot with linear regression analysis of the individual patient data points for NGAL in relation to creatinine showed a strongly significant positive relationship. The coefficient of determination (R^2^ value) was 0.70. This means that, each unit increase of NGAL will influence 70.0% of the increase change in the creatinine levels. NGAL was significant negatively related with MDRD equation (*p* < 0.0001), Cockroft-Gault equation (*p* < 0.0001) and CKD-EPI creatinine equation (*p* < 0.0001). The coefficients of determination (R^2^ = value) were 0.42, 0.55 and 0.27 which means that, each unit decrease of NGAL influences 42.0%, 55.0% and 27.0% increase change in values of MDRD equation, Cockroft-Gault equation and CKD-EPI creatinine equations.

The findings of this current study was comparable to the work of David Bolignano et al. [21] in the use of NGAL in prediction of CKD.

## Conclusion

The results of this current study indicate that pNGAL is a better indicator of kidney impairment in the early stages of CKD as compared with serum Creatinine in general hypertensive population. NGAL showed good cut-off point in diagnosing renal impairment with good sensitivity and specificity compared to creatinine. Also, hypertension is a common cause of CKD and hence patients with high blood pressure are at increased risk of loss of kidney function and development of CKD. Individuals with high BP should therefore be carefully evaluated for the presence of CKD especially those with decreased GFR using the NGAL biomarker. It is important to look into the significance of pNGAL as a biomarker for the early detection of kidney failure among other populations and for the differential diagnosis of renal disease etiology in other cardiometabolic illnesses.

## Data Availability

Data and materials for the study are available upon request from the corresponding authors.

## Abbreviations

AKI: Acute Kidney Injury
CKD: Chronic Kidney Disease
BP: Blood Pressure
BUN: Blood Urea Nitrogen
CG: Cockroft Gault
CKD-EPI: Chronic Kidney Disease- Epidemiology Collaboration
Ccr: Creatinine Clearance
eGFR: Estimated Glomerular Filtration Rate
NGAL: Neutrophil Gelatinase Associated Lipocalin
MDRD: Modification of Diet in Renal Disease

## References

[1] Palevsky PM, Liu KD, Brophy PD, Chawla LS, Parikh CR, Thakar CV (2013). KDOQI US commentary on the 2012 KDIGO clinical practice guideline for acute kidney injury. Am J Kidney Dis.

[2] Levey AS, Coresh J, Balk E, Kausz AT, Levin A, Steffes MW (2003). National Kidney Foundation practice guidelines for chronic kidney disease: evaluation, classification, and stratification. Ann Intern Med.

[3] Couser WG, Remuzzi G, Mendis S, Tonelli M (2011). The contribution of chronic kidney disease to the global burden of major noncommunicable diseases. Kidney Int.

[4] Abd ElHafeez S, Bolignano D, D’Arrigo G, Dounousi E, Tripepi G, Zoccali C (2018). Prevalence and burden of chronic kidney disease among the general population and high-risk groups in Africa: a systematic review. BMJ Open.

[5] Stanifer JW, Jing B, Tolan S, Helmke N, Mukerjee R, Naicker S (2014). The epidemiology of chronic kidney disease in sub-Saharan Africa: a systematic review and meta-analysis. Lancet Glob Heal.

[6] James MT, Hemmelgarn BR, Tonelli M (2010). Early recognition and prevention of chronic kidney disease. Lancet.

[7] Sanyaolu A, Okorie C, Annan R, Turkey H, Akhtar N, Gray F (2018). Epidemiology and management of chronic renal failure: a global public health problem. Biostat Epidemiol Int J.

[8] Wu L, Gao Y, Zhang S, Fang Z (2019). The effects of breviscapine injection on hypertension in hypertension-induced renal damage patients: a systematic review and a meta-analysis. Front Pharmacol.

[9] Cappuccio FP, Micah FB, Emmett L, Kerry SM, Antwi S, Martin-Peprah R (2004). Prevalence, detection, management, and control of hypertension in Ashanti. West Africa Hypertension.

[10] Tantisattamo E, Molnar MZ, Ho BT, Reddy UG, Dafoe DC, Ichii H (2020). Approach and management of hypertension after kidney transplantation. Front Med.

[11] Osafo C, Mate-Kole M, Affram K, Adu D (2011). Prevalence of chronic kidney disease in hypertensive patients in Ghana. Ren Fail.

[12] Raman M, Middleton RJ, Kalra PA, Green D (2017). Estimating renal function in old people: an in-depth review. Int Urol Nephrol.

[13] Vijayan A, Faubel S, Askenazi DJ, Cerda J, Fissell WH, Heung M (2016). Clinical use of the urine biomarker [TIMP-2]×[IGFBP7] for acute kidney injury risk assessment. Am J Kidney Dis.

[14] Kadry Y, Abd Allah AA, Ali A, Omar H (2015). NEutrophil gelatinase–associated LIPOCALIN (NGAL) as an early biomarker of acute kidney injury in hepatic patients. Zagazig Univ Med J.

[15] Mitsnefes MM, Kathman TS, Mishra J, Kartal J, Khoury PR, Nickolas TL (2007). Serum neutrophil gelatinase-associated lipocalin as a marker of renal function in children with chronic kidney disease. Pediatr Nephrol.

[16] Buonafine M, Martinez-Martinez E, Jaisser F (2018). More than a simple biomarker: the role of NGAL in cardiovascular and renal diseases. Clin Sci.

[17] Asare-Anane H, Ofori EK, Yeboah FA, Tagoe EA, Bani SB, Bawah AT (2013). Primary hypogonadism in Ghanaian men with type 2 diabetes mellitus. Cell.

[18] Kumela Goro K, Desalegn Wolide A, Kerga Dibaba F, Gashe Fufa F, Wakjira Garedow A, Edilu Tufa B (2019). Patient awareness, prevalence, and risk factors of chronic kidney disease among diabetes mellitus and hypertensive patients at Jimma University medical center. Ethiopia. Biomed Res Int.

[19] Mallat SG, Al Kattar S, Tanios BY, Jurjus A (2016). Hyperuricemia, hypertension, and chronic kidney disease: an emerging association. Curr Hypertens Rep.

[20] Gharishvandi F, Kazerouni F, Ghanei E, Rahimipour A, Amirrsouli H, Nasiri M. Assessment of Neutrophil gelatinase-associated lipocalin (NGAL) as an early biomarker for detection of renal impairment in hypertensive patients. Arch Adv Biosci. 2014;5(1). ISSN 2008-4978.

[21] Bolignano D, Coppolino G, Campo S, Aloisi C, Nicocia G, Frisina N (2008). Urinary neutrophil gelatinase-associated lipocalin (NGAL) is associated with severity of renal disease in proteinuric patients. Nephrol Dial Transplant.

[22] Malyszko J, Bachorzewska-Gajewska H, Malyszko JS, Pawlak K, Dobrzycki S (2008). Serum neutrophil gelatinase-associated lipocalin as a marker of renal function in hypertensive and normotensive patients with coronary artery disease. Nephrology.

[23] Wagener G, Jan M, Kim M, Mori K, Barasch JM, Sladen RN (2006). Association between increases in urinary neutrophil gelatinase–associated lipocalin and acute renal dysfunction after adult cardiac surgery. J Am Soc Anesthesiol.

[24] Song E, Fan P, Huang B, Deng H, Cheung BMY, Félétou M (2014). Deamidated lipocalin-2 induces endothelial dysfunction and hypertension in dietary obese mice. J Am Heart Assoc.

[25] Chen J, Wildman RP, Gu D, Kusek JW, Spruill M, Reynolds K (2005). Prevalence of decreased kidney function in Chinese adults aged 35 to 74 years. Kidney Int.

[26] Kurella M, Lo JC, Chertow GM (2005). Metabolic syndrome and the risk for chronic kidney disease among nondiabetic adults. J Am Soc Nephrol.

[27] Locatelli F, Pozzoni P, Del Vecchio L (2006). Renal manifestations in the metabolic syndrome. J Am Soc Nephrol.

[28] Pinto-Sietsma S-J, Navis G, Janssen WMT, de Zeeuw D, Gans ROB, de Jong PE (2003). A central body fat distribution is related to renal function impairment, even in lean subjects. Am J Kidney Dis.

[29] Levey AS, Stevens LA, Schmid CH, Zhang Y, Castro AF, Feldman HI (2009). A new equation to estimate glomerular filtration rate. Ann Intern Med.

